# Robust Quantification of Live-Cell Single-Molecule Tracking Data for Fluorophores with Different Photophysical Properties

**DOI:** 10.1021/acs.jpcb.4c01454

**Published:** 2024-06-11

**Authors:** Amy N. Moores, Stephan Uphoff

**Affiliations:** Department of Biochemistry, University of Oxford, South Parks Rd, Oxford OX1 3QU, U.K.

## Abstract

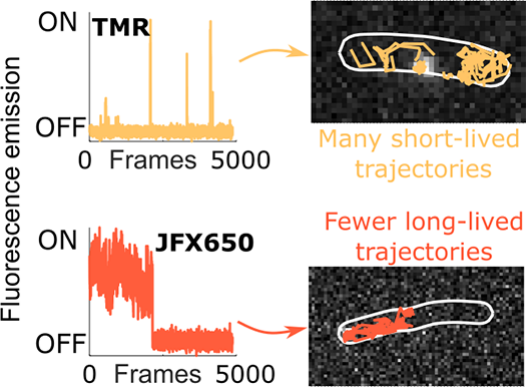

High-speed single-molecule tracking in live cells is becoming an increasingly popular method for quantifying the spatiotemporal behavior of proteins *in vivo*. The method provides a wealth of quantitative information, but users need to be aware of biases that can skew estimates of molecular mobilities. The range of suitable fluorophores for live-cell single-molecule imaging has grown substantially over the past few years, but it remains unclear to what extent differences in photophysical properties introduce biases. Here, we tested two fluorophores with entirely different photophysical properties, one that photoswitches frequently between bright and dark states (TMR) and one that shows exceptional photostability without photoswitching (JFX650). We used a fusion of the *Escherichia coli* DNA repair enzyme MutS to the HaloTag and optimized sample preparation and imaging conditions for both types of fluorophore. We then assessed the reliability of two common data analysis algorithms, mean-square displacement (MSD) analysis and Hidden Markov Modeling (HMM), to estimate the diffusion coefficients and fractions of MutS molecules in different states of motion. We introduce a simple approach that removes discrepancies in the data analyses and show that both algorithms yield consistent results, regardless of the fluorophore used. Nevertheless, each dye has its own strengths and weaknesses, with TMR being more suitable for sampling the diffusive behavior of many molecules, while JFX650 enables prolonged observation of only a few molecules per cell. These characterizations and recommendations should help to standardize measurements for increased reproducibility and comparability across studies.

## 1 Introduction

Single-molecule localization microscopy has deepened our understanding of the structural organization of cells. Techniques such as stochastic optical reconstruction microscopy (STORM)^1^ and (fluorescence) photoactivated localization microscopy ((F)PALM)^2,3^ were originally performed on fixed cells, enabling the detection of fluorescently labeled proteins at localization precisions down to approximately 10 nm. These methods ultimately allow image reconstructions of cellular structures at resolutions beyond the diffraction limit (<250 nm). Single-molecule localization microscopy is made possible by the specific labeling of molecules of interest with fluorescent proteins or dyes that have unique photophysical properties, allowing fluorescence emission to be activated or reversibly deactivated by performing different illumination strategies; these fluorophores are often referred to as “photoactivatable” or “photoswitchable”, or more generally as “photocontrollable”.^4,5^ One popular option for single-molecule studies is the use of fluorophores that have a distinct fluorescence “on” state and a longer-lived “off” state. Stochastic and reversible switching between these two states by each individual fluorophore allows molecules to be localized for a short time while in the “on” state, before they randomly fall to the “off” state. Crucially, because the “off” state is longer-lived than the “on” state, the likelihood of adjacent molecules emitting simultaneously within the same cell is low, providing the sparse-emitter conditions required for imaging single molecules.^6^ A localization algorithm is applied to determine the centroid of the isolated point spread function for each molecule, and a super-resolved image is generated from the combined map of localizations that have occurred over time.

**Figure 1:**
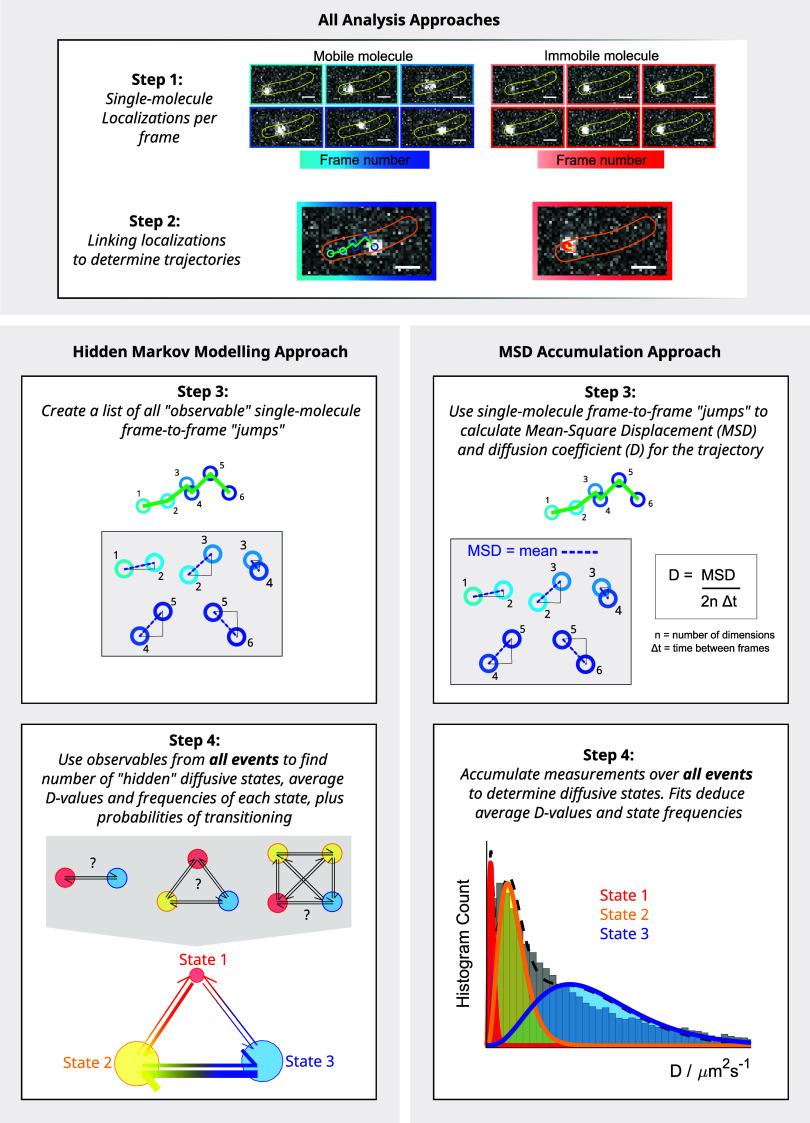
Schematic describing the analysis of single-molecule tracking data to quantify subpopulations of molecules with different diffusion coefficients. Two examples of MutS-Halo-TMR single-molecule fluorescence events are shown (associated with mobile and immobile molecules), which appeared in the same cell at different time points in a movie. We show how localizations are linked across frames to determine the MSD and subsequent diffusion coefficient estimates from each trajectory. Repeat observations are made to accumulate diffusion coefficient measurements. In this example, we see three diffusive states in the diffusion coefficient histogram: fast-diffusing (blue), immobile (red), and a slowly diffusing state (yellow). Relative abundances of the three subpopulations could be quantified by fitting the histogram. In HMM analysis, diffusive states are inferred from the molecule displacements between successive frames without averaging over trajectories, and the state occupancies are estimated from the transition probabilities between the states.

Beyond the study of fixed molecular structures, this approach can be generalized for imaging live cells, where the single-molecule localization strategy helps to visualize the movement of individual proteins inside cells.^7^ The utility of the approach is exemplified well for DNA-binding proteins, which typically exist in mobile and immobile states, corresponding to molecules that either search for, or dwell at, DNA-binding sites, respectively.^8−15^Figure 1 shows how this single-molecule tracking technique works in practice. Where photoswitchable or photoactivatable fluorophores are used, they can provide short glimpses of isolated molecules as they move through a cell. The precise locations of these appearances are determined within each frame of an acquired movie, and a tracking algorithm is applied that links the coordinates to reconstruct the pathway that each molecule has taken through the cell. For photoswitchable dyes, it is possible to see multiple emission events from the same molecule throughout the duration of the acquisition. Although the average duration of tracks is short (typically <10 frames per track), very large data sets are easily generated (often comprising many thousands of tracks per movie), which necessitates the use of automated data analysis algorithms.

Single-molecule tracking data sets evidently contain a wealth of information about the function of the labeled molecules, but it can be very challenging to extract this information in a reliable and efficient way. A simple and informative metric is the diffusion coefficient, which characterizes the mobility of molecules that move by random Brownian motion. Each observed trajectory provides one measurement of the diffusion coefficient of the molecule of interest, which is calculated from the average squared distance between successive localizations in a track (i.e., the mean squared displacement, MSD). Commonplace analysis methods accumulate all measurements of the diffusion coefficient into a histogram (such as that in Figure 1) or a cumulative distribution, from which subpopulations of molecules with different mobilities can be identified and quantified (using various types of distribution fitting and parameter estimation approaches).^8,13,16−20^ While these approaches interpret tracking data from the viewpoint of entire diffusive subpopulations, we could equivalently interpret the data from the viewpoint of each individual molecule: individual molecules should not have a memory of their previous states, and they should be able to transition between diffusive states repeatedly, on an unknown time scale. Therefore, the percentage of time that an individual molecule spends in each diffusive state should match the overall percentage of molecules that reside in each of the states at any one time. This ergodic interpretation is utilized by Hidden Markov Modeling (HMM) and related approaches^21−27^ that allow diffusive state lifetimes and transition rates to be inferred from single-molecule tracking datasets, despite the fact that directly observing transitions between states during the “on”-time of a fluorophore is rare (Figure 1). Both methods, MSD and HMM analyses, have been successfully applied to study a variety of proteins and other biomolecules that change their intracellular mobility when they perform their functions.^8,11,20,28−32^ Although this study focuses on application of single-molecule tracking in bacterial cells, the findings are also relevant for applications of the method to eukaryotic and archaeal cells.^33,34^

The advent of genetically encoded protein tags (e.g., HaloTag^35^ and SnapTag^36^) has enabled the use of bright synthetic fluorophores, which can readily permeate live cells and specifically label proteins of interest for super-resolution imaging. A popular choice for single-molecule experiments is tetramethylrhodamine (TMR), which is a bright dye that exhibits reversible photoswitching with an average on-state duration of <1 s in live cells.^37^ Owing to phenomenal advances in the chemistry of fluorophores, many new variants of TMR and related fluorophores with improved photophysical properties have been synthesized.^38−40^ Dyes with superior photostability promise longer-lived on-states,^41^ which, when used for single-molecule tracking, could provide an exciting opportunity to follow the movement of a protein of interest for several minutes. The long-lived emission made possible with these new fluorophores makes direct observations of transitions between diffusive states more likely. This motivated us to test whether the same quantitative results are obtained when using a long-lived photostable dye compared to a short-lived photoswitching dye when labeling the same protein of interest and performing single-molecule tracking. We explore whether data acquisition and analysis procedures are robust to the use of different fluorophores or whether any new biases are introduced by altering the photophysical properties of the fluorophore. Addressing these questions will aid comparison and validation of single-molecule tracking results across studies that implemented different fluorescent dyes and also help the user to make the right choice of fluorophore depending on the experiment type.

Here, we chose the well-characterized dye TMR and new photostable dye JFX650,^39^ created by incorporating deuterium into the auxochrome of rhodamine. The dyes differ in color (excitation/emission maxima 557/576 nm for TMR and 650/667 nm for JFX650) and show very different photophysical behavior, making them ideal test cases for this study. We analyzed the diffusion characteristics of the DNA repair enzyme MutS in live *Escherichia coli* bacteria for this investigation. Single-molecule tracking has previously been applied to study MutS in *E. coli*(42) and *B. subtilis*.^31^ We describe the key differences required for the acquisition of single-molecule tracking data between the two dyes, assess the comparability of MSD and HMM data analysis methods, and discuss strategies to minimize analysis biases associated with differences in the fluorophore photostability.

## 2 Methods

### 2.1 Preparation of *E. coli* Cells with Fluorescently Labeled MutS for Single-Molecule Tracking

We chose the *E. coli* DNA mismatch repair (MMR) enzyme MutS as a test protein for comparison of TMR and JFX650 fluorophores in single-molecule tracking experiments. MutS is essential for the repair of misincorporated bases during DNA replication. MutS binds to DNA mismatches and initiates a cascade of reactions involving multiple enzymes to excise and resynthesize the stretch of DNA including the mismatch.^43−45^

To image MutS, we utilize a translational fusion of MutS to the HaloTag.^35^ The tag is attached to MutS via a flexible linker of 11 amino acids, and the construct is expressed from the native chromosomal locus of *E. coli* strain AB1157 (see further details in Supplementary S.1.1).

We labeled MutS-Halo using TMR (Promega UK) or JFX650 (Janelia Farm) using a protocol described previously,^37^ which includes a short incubation period and a series of wash steps to remove nonspecifically bound fluorescent molecules. Briefly, cells are grown on LB plates and a colony is isolated, grown in LB media for 4 h before being diluted (1 in 2000) in M9 media (see recipe in Supplementary S.1.2), and grown overnight. The following day, the overnight culture is diluted 1 in 50 in M9 media and grown to an OD_600_ of approximately 0.1 (to early exponential phase, ca. 2 h growth time). HaloTag TMR ligand and HaloTag JFX650 ligand are diluted from DMSO stock solutions to a final concentration of 50 μM in Milli-Q water. Cells are centrifuged to form a pellet and are then resuspended in 100 μL of M9 media to form a concentrated culture to which, for the majority of experiments, 5 μL (TMR) or 0.5 μL (JFX650) of the 50 μM dye solution is added and incubated at room temperature for 30 min. Three repeat washes are performed with M9 media by a series of centrifugation and resuspension steps. After washing, cells are recovered for 30 min by shaking at 37 °C. A final wash is performed following recovery before cells are concentrated and spotted onto M9 agarose pads.

Agarose pads are made by adding molecular biology grade agarose (Bio-Rad) to M9 media (to 1% concentration) and gently heating in a microwave. Approximately 1 mL of M9 agarose is then pipetted onto borosilicate coverglass (#1.5 thickness, VWR International), sandwiched by a second coverglass on top, and left to solidify. Immediately before imaging, the top coverglass is removed, cells are spotted directly onto the agarose pad, and a new coverglass is placed on top of the cells. Coverglass were treated in a plasma chamber (Inseto PE-50) to remove fluorescent background particles before use.

The differences in labeling concentration for the TMR and JFX650 experiments were governed by the different photophysical properties of TMR and JFX650 dye molecules (see Results and Discussion Section 3.1). For the experiments in results section 3.1, we used equivalent labeling concentrations of 2.5 μM within 100 μL cell culture for both the TMR and the JFX650 imaging to initially compare bleaching kinetics. For all other experiments, we used a final concentration within 100 μL of cell culture of 2.5 μM for TMR and a lower concentration of 0.25 μM for JFX650 to adjust the labeling density and maintain sparse-emitter conditions during single-molecule imaging.

### 2.2 Single-Molecule Tracking Acquisition

For single-molecule imaging, we use an inverted optical microscope with TIRF capabilities (see Supplementary section S.1.3 for further details regarding microscope configuration). Cells were first illuminated with an LED condenser to capture a transmitted light image (exposure time of 30 ms), which aids cell segmentation during the analysis of trajectories. Following this, cells were illuminated with 561 nm (for TMR-labeled MutS-Halo) or 640 nm lasers (for JFX650-labeled MutS-Halo). In both cases, the power density at the sample plane is approximately 1 Wcm^–2^.

For TMR, most MutS-Halo-TMR molecules are initially in the fluorescent-on state, so we recorded movies after waiting for 30–60 s following laser illumination to allow photoswitching cycles to commence and sparse-emitter conditions to be reached. As discussed below (Results and Discussion Section), JFX650 does not show reversible photoswitching, so we recorded movies immediately from the moment of laser excitation. The lower labeling concentration used for JFX650 means that sparse-emitter conditions are achieved from the start of the movie.

For all single-molecule acquisitions, we recorded movies of 5000 frames with an exposure time of 30 ms/frame, giving an interval of 30.475 ms between successive frames of the movies due to the camera readout time. Typically, 6–8 well-spaced regions of each sample were imaged, providing data for >1000 cells per session. At least 3 independent imaging sessions were performed for each condition. We note that samples usually have a subpopulation of dead cells in which TMR and JFX650 dyes bleach slowly and proteins appear completely immobilized. We characterized these cells as dead based on a live–dead fluorescent cell stain (Supplementary Figure S2). These cells were excluded from further analysis.

### 2.3 Single-Molecule Tracking Analysis

Single-molecule imaging data were processed and analyzed as follows. First, single molecules are localized using a phasor localization detection algorithm^46^ by setting a minimum intensity threshold for point spread function detection. Following the localization step, detected molecules are linked together to form single-molecule tracks if the following criteria are met: (1) the distance between localizations in subsequent frames is less than 8 pixels and (2) the localizations arise from the same cell based on the segmentation masks from the transmitted light image. In addition, the tracking algorithm incorporates a one-frame memory parameter: that is, a localization is allowed to be missing from 1 frame of the movie and the two localizations on either side of the missed frame will still be linked together if criteria 1 and 2 are met. The result is a list of trajectories that can be used in either of the two analysis approaches: MSD or HMM analysis. For MSD analysis, each linked trajectory provides one measurement of the diffusion coefficient; for this, the first 5 frames (4 frame-to-frame steps) of the trajectory are used to calculate the MSD, which can then be used to determine the diffusion coefficient via the calculation shown in Figure 1. All measurements of the diffusion coefficient from one imaging session (i.e., for the same condition) are then accumulated into a single histogram, to which we fit a mixture of probability density functions according to the number of diffusive states believed to be present in the data.^13^ Each of the diffusive state probability functions take the form of a Gamma distribution^16^:
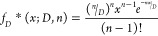
where *n* is the number of steps the MSD is being calculated over, *D* is the average diffusion coefficient, and *x* is the number of individual measurements made (see the Supporting Information, S.1.4, for further details). The fit parameters yield estimates of the diffusion coefficient and relative occupancy for each state. Each imaging session is treated as an independent measurement of the diffusion coefficient values and state occupancies. For HMM analysis, we used the analysis tool vbSPT,^23^ which identifies the minimum number of diffusive states consistent with the data and an estimate for the diffusion coefficient of each state. It also provides the transition probabilities between states and relative state occupancies.

## 3 Results and Discussion

### 3.1 Photophysical Properties of JFX650 and TMR Necessitate Different Data Acquisition Approaches

The photophysical properties of fluorophores are key determinants for the accurate localization of single molecules and the successive linking of their coordinates to form single-molecule trajectories. Some of these photophysical properties and how they affect potential biases in the estimation of diffusion coefficients are already well-characterized. For example, prolonged on-state lifetimes increase the emitter density, which hinders reliable molecule tracking.^47^ Additionally, differences in fluorophore brightness affect the localization precision and therefore the apparent diffusion coefficients and the ability to distinguish between different diffusive states.^48^ Fast-moving molecules undergo larger displacements within each exposure, leading to “motion blur”^49^ (also termed “dynamic error”^50,51^); this effect causes an inherently dimmer appearance within each frame, and therefore, the fast-moving molecules are more likely to drop below the intensity threshold for localization, which may cause an underestimation of mobile state occupancies, as previously reported.^47^ To ensure that these inherent biases affect experiments with TMR and JFX650 to the same degree (so as not to affect the overall comparison), we designed our experimental approach with the aim of maintaining similar localization precisions and emitter densities for both fluorophores.

We first adjusted the 561 and 640 nm laser intensities for excitation of TMR and JFX650, respectively, to provide comparable fluorescent brightness and therefore localization error (see Supplementary Figure S1 and corresponding details in Supplementary section S.2). This ensures that biases in the quantification of diffusion coefficients and state occupancies due to localization error and missed localizations are similar for the two dyes.

**Figure 2:**
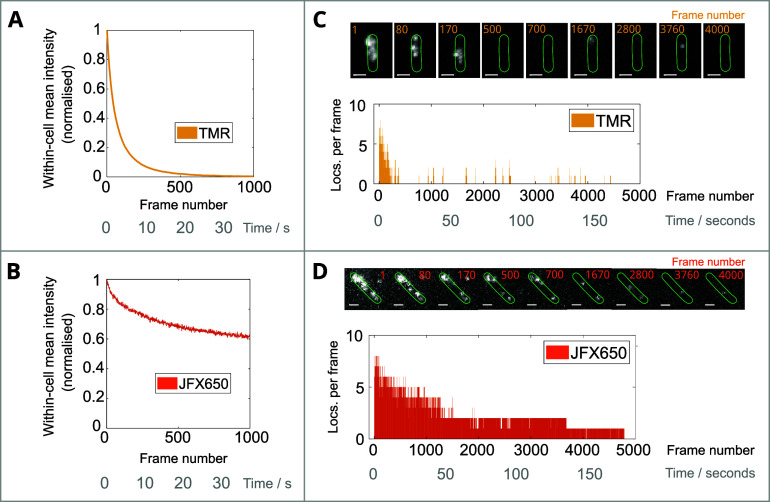
Comparison of TMR- and JFX650-labeled MutS-HaloTag photobleaching. (A, B) Mean intensity of cells with (A) TMR-labeled or (B) JFX650-labeled MutS-Halo for 1000 frames (≈35 s) from laser turn-on. The labeling concentrations used for both TMR and JFX650 were 2.5 μM. The mean background intensity outside cells was subtracted from the intensity values of all pixels within the segmented cell masks and averaged across 506 and 1477 cells. Curves were normalized by the maximum value (from 3 and 7 movies) for TMR and JFX650, respectively. (C, D) The number of localizations within one representative cell expressing (C) TMR-labeled or (D) JFX650-labeled MutS-HaloTag for a duration of 5000 frames (≈174 s) from laser turn-on. Snapshots of the movie with segmented cell masks at the indicated frames are shown. We note that the high emitter density prior to photobleaching will prevent accurate localization and likely leads to an underestimation of the true number of localizations per frame at the start of the acquisitions. Scale bars 1 μm.

Next, we determined the photoswitching and bleaching kinetics of JFX650 and TMR from the average intensity across a population of cells (Figure 2A,B) and from the number of localizations detected in a single representative cell (Figure 2C,D). For this experiment, we used the same labeling concentration for TMR as for JFX650 (see discussion in the Methods section); we note that the relatively high emitter density used may lead to an underestimation of the true localization number for both TMR and JFX650 at the start of the acquisition periods. Nevertheless, for the TMR case, the results portray a rapid decay in cellular fluorescence and localization numbers following laser turn-on. The sparse-emitter condition of fewer than 1 localization per cell is reached within 500 frames. The majority of proceeding frames have zero localizations detected, interspersed with brief but distinct periods where single TMR molecules become fluorescent again and generate a track of localizations. Conversely, JFX650 is much more photostable than TMR and the irreversible photobleaching of individual molecules can be seen as a stepwise drop in localization numbers for the single cell. We note that the intermittent peaks and troughs in localization number are not attributed to photoswitching for JFX650, but that they can be traced back to frames with either false localizations or frames where molecules are crossing paths within the cell and cannot be individually isolated. Other than rare sporadic localizations due to background noise, we see no repeat activation from the JFX650 molecules.

For single-molecule tracking with TMR, the sustained photoswitching with a long off-state after the initial rapid deactivation period provides sparse-emitter conditions over prolonged periods of time even when the total number of fluorescently labeled molecules is very high. However, for JFX650, the high photostability results in a high emitter density, and the lack of repeated photoswitching means that tracks should ideally be recorded from the moment of laser turn-on. To achieve sparse-emitter conditions for JFX650, we therefore lowered the labeling density using 10-fold reduced concentration of dye compared to TMR for all subsequent single-molecule tracking experiments.

### 3.2 Comparison of TMR and JFX650 Single-Molecule Tracking Using HMM Analysis

**Figure 3:**
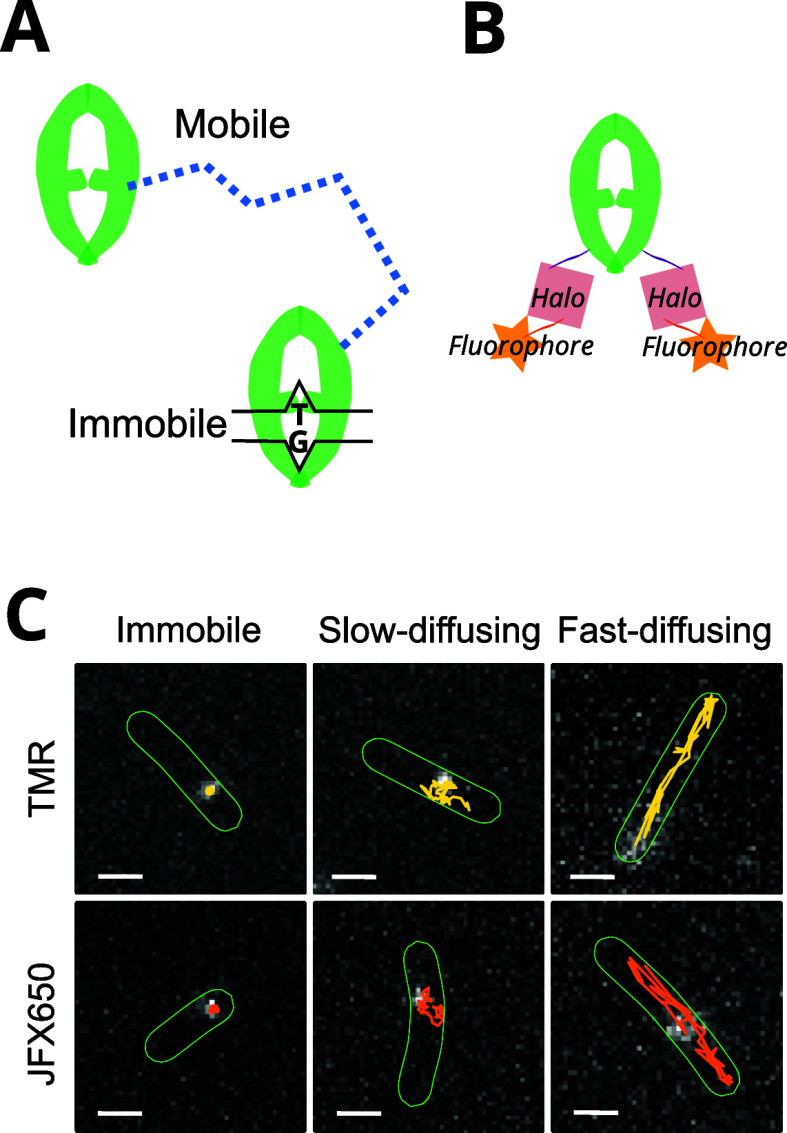
Single-molecule tracking of MutS-Halo. (A) Schematic of MutS undergoing target search and binding a DNA mismatch, corresponding to mobile and immobile diffusive states, respectively. (B) Schematic of a fluorescently labeled MutS-Halo dimer. (C) Example MutS-Halo trajectories for TMR (yellow) or JFX650 (orange) display three distinct diffusive states: immobile, slow-diffusing, and fast-diffusing. All example trajectories have the same duration of 40 frames. Trajectories are overlaid onto a fluorescence image corresponding to the final position of the molecule, on which the cell segmentation masks are also displayed (green). Scale bars 1 μm.

We first characterized the single-molecule trajectories from each set of experiments using HMM. The HMM approach assumes that there is an observable process (in this case, the measured frame-to-frame displacement of MutS-Halo molecules) whose state depends on the outcomes of a second hidden process (in this case, the possible transitions between different diffusive states of MutS-Halo); the aim is to learn about the state of the hidden process through measurements of the observable process. We utilized the HMM software vbSPT,^23^ which requires a user-defined estimate for the number of diffusive states present in the data. We performed several iterations of vbSPT analysis on our single-molecule tracking data, varying the maximum number of diffusive states between 2 and 6 (see Figure S3). We chose the 3-state model as the one that best represents MutS-Halo diffusive motion, providing an immobile state, slowly moving state, and faster mobile state. We observed clear examples of trajectories exhibiting these diffusion types in both the TMR- and JFX650-labeled MutS-Halo experiments (Figure 3C). Although the exact biological functions of these diffusive states remain unclear, we note that there are several potential reasons for their existence. *E. coli* MutS binds DNA as a homodimer (Figure 3A,B), which is known to slide on DNA and changes conformations during the DNA mismatch search and repair process.^52^ Furthermore, MutS also forms tetrameric complexes in solution.^53^ The different oligomeric states and DNA-binding modes may give rise to multiple molecular subpopulations with different diffusion coefficients. We also speculate that molecules may display different diffusive behaviors due to heterogeneity of the cellular environment, e.g., diffusing within or outside the nucleoid.^14,54,55^

**Figure 4:**
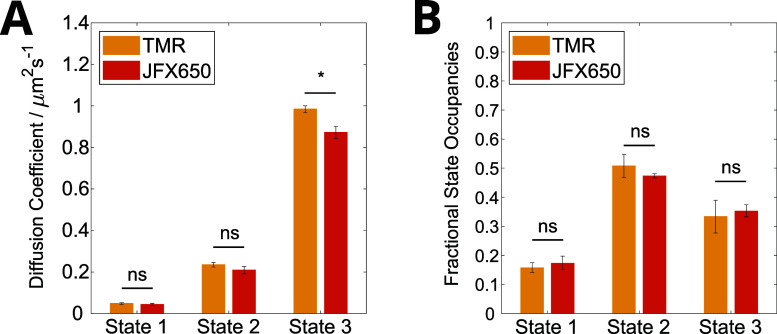
Comparison of TMR- and JFX650-labeled MutS-Halo diffusive state analysis using HMM. Shown are the diffusion coefficient values (A) and the state occupancies (B) for the 3-state model. Error bars show the standard error of the mean across 3x (TMR) and 6x (JFX650) independent experimental repeats, and bar heights are weighted means according to the total number of trajectories per repeat.

Using the HMM analysis with a 3-state diffusion model, the output diffusion coefficients and occupancies of the states were in excellent agreement between TMR and JFX650 (Figure 4). Only the fast-diffusing state had a slightly slower diffusion coefficient estimate for MutS-Halo-JFX650 compared to that of MutS-Halo-TMR (0.87 vs 0.99 μm^2^s^–1^). This difference was subtle but significant and could be caused by genuine differences in the mobility of MutS-Halo due to differences in the physical properties of the attached dyes such as their charge and size. Nevertheless, our results indicate that the measurement of diffusion coefficients and the relative frequency of each diffusive state via single-molecule tracking are largely reliable when HMM analysis is utilized, despite the fact that dyes with very different photophysical properties were used.

### 3.3 Comparison of TMR and JFX650 Single-Molecule Tracking Using MSD Analysis

**Figure 5:**
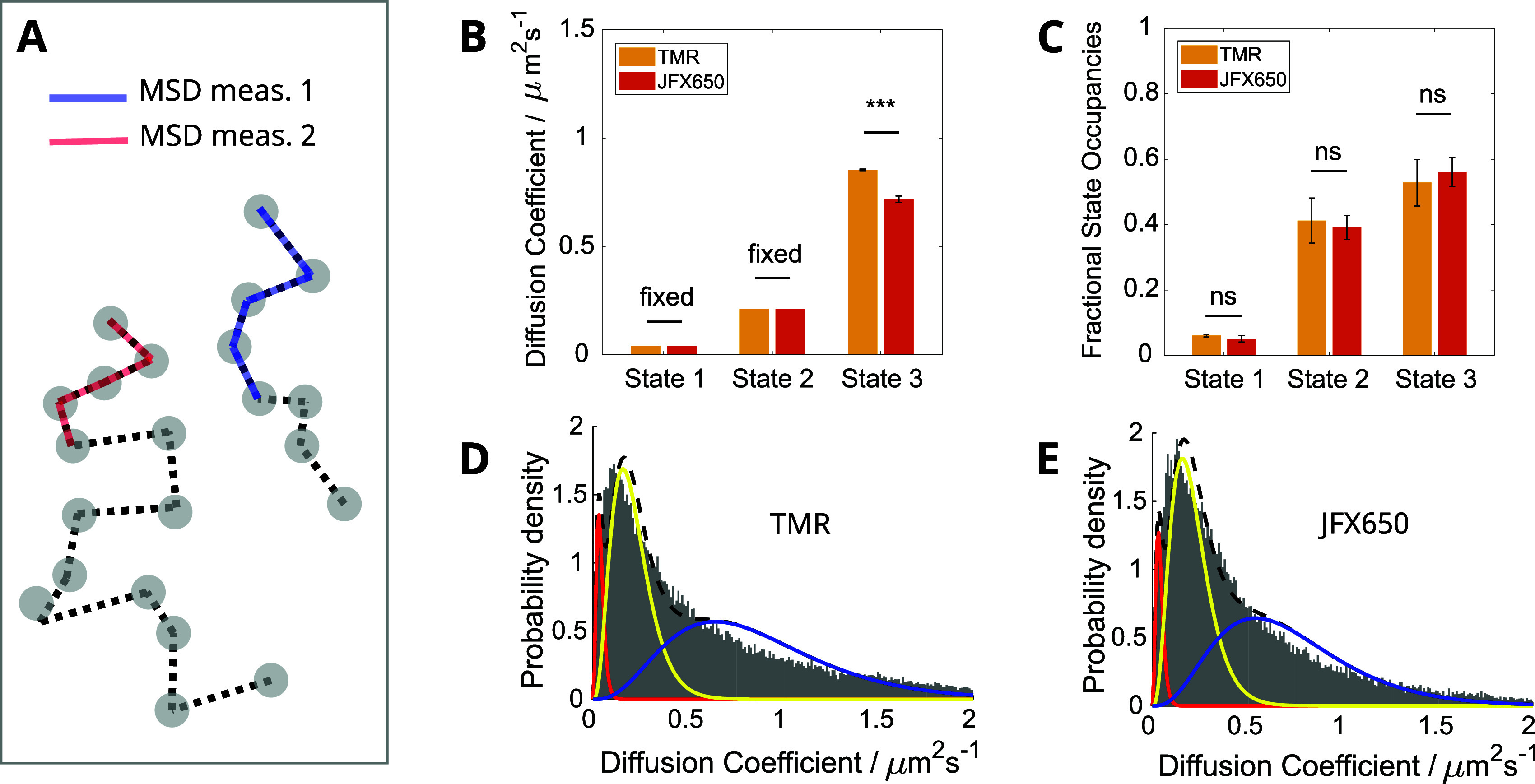
Comparison of TMR- and JFX650-labeled MutS-Halo diffusive state analysis using the MSD calculation approach. (A) Schematic showing the frames over which the MSD is measured for each example trajectory. (B, C) Estimated diffusion coefficients and state occupancies for the 3-state model. Error bars show the standard error of the mean across 3x (TMR) and 6x (JFX650) independent experimental repeats, and bar heights are weighted means according to the total number of trajectories per repeat. (D, E) Averaged diffusion coefficient histograms from each experiment repeat for TMR (D) and JFX650 (E). The fitted probability distribution curves are shown for the immobile (red), slowly diffusing (yellow), and fast-diffusing (blue) states.

Unlike HMM, MSD analysis involves computing the average molecular displacement to estimate the diffusion coefficient for each track. Figure 5A portrays these calculations; for each trajectory, we estimate the MSD based upon the first 5 frames (or first 4 frame-to-frame steps) only. With this process, each trajectory provides one diffusion coefficient measurement. Because the number of frames over which the calculations are made is fixed, we can estimate the diffusion properties for the 3-state model by fitting a mixture of probability density distributions to a histogram of accumulated diffusion coefficient measurements^13^ (see Figure 1). We utilized the insights from the HMM analysis to aid the fitting by fixing the diffusion coefficients of the immobile and slowly diffusing states (to 0.04 and 0.21 μm^2^s^–1^, respectively) while leaving the fast-diffusing population unconstrained (Figure 5D,E). The state occupancies show no significant difference between TMR- and JFX650-labeled MutS-Halo (Figure 5C). We again see a small but significant difference in the diffusion coefficient for the fast-diffusing state, indicating that this is likely a genuine difference between the MutS-Halo molecules labeled with the two different fluorophores (Figure 5B). These results again suggest that measurements of the diffusion coefficients and the relative frequencies of each diffusive state are largely consistent, regardless of the fluorophore used for single-molecule tracking.

### 3.4 State Occupancy Biases in MSD Analysis Are Amplified for Photostable Fluorophores

Although the choice of fluorophore seems to make little difference to the diffusion analysis, our results suggest that the choice of analysis method does. Comparing the results of HMM and MSD analysis methods revealed that the MSD approach consistently estimated a lower occupancy of the immobile state compared to HMM (*p* = 0.0059, *p* = 0.00018, for TMR and JFX650, respectively, see Supplementary Figure S5). Probing further, we noticed that cells with MutS-Halo-JFX650 often contained far more trajectories than expected given the low labeling concentration and lack of reversible photoswitching for this dye. This led us to suspect that trajectories arising from single fluorescence events were often partitioned into multiple subtrajectories (which we call “inadvertent splitting”), and that this splitting was more frequent for mobile than immobile molecules. Consequently, the apparent frequency of molecules in a mobile state is over-represented in histograms, leading to an overestimation of the mobile state occupancies; this presents as a corresponding underestimation of the immobile state occupancy for the MSD analysis approach.

**Figure 6:**
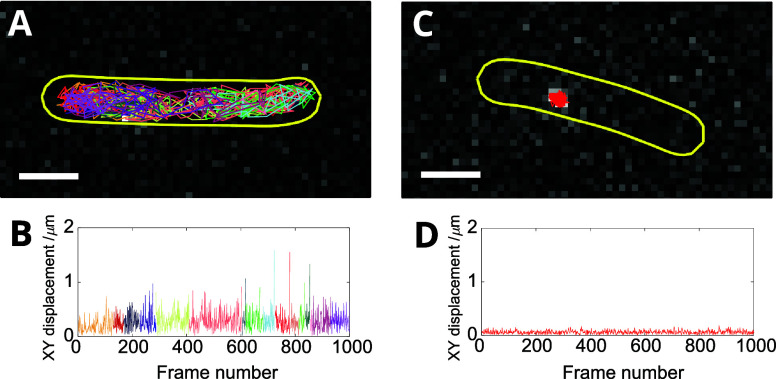
Inadvertent splitting of trajectories causes overcounting bias of mobile molecules. (A) Example MutS-Halo-JFX650 trajectory (1000 frames provided) of one mobile molecule, overlaid on a single frame of the movie. The analysis algorithm has failed to correctly link together all localizations arising from the same molecule, resulting in 19 separate subtrajectories, each plotted with a random color. (B) The corresponding measured molecular frame-to-frame displacement plotted as a function of frame number. Line colors correspond to those of the individual tracks plotted in panel A. (C, D) Example MutS-Halo-JFX650 trajectory (1000 frames provided) and displacements of one immobile molecule. The analysis algorithm has correctly linked together all localizations resulting in a single trajectory. Segmented cell outlines are depicted (yellow). Scale bars 1 μm.

We found evidence of this phenomenon by inspecting cells that visibly contained just a single fluorescent MutS-Halo-JFX650 molecule throughout the movie. Figure 6 shows two different cells, in which a single mobile molecule and single immobile molecule were observed, respectively (Figure 6A,B;C,D). Both trajectories have been cut to the same duration of 1000 frames for visualization; however, for the same window of observation, localization and tracking analysis resulted in 19 subtrajectories for the cell with a single mobile molecule, whereas the immobile molecule was successfully retained as one single track. In effect, this causes a 19-fold overestimation of the mobile state occupancy for this particular pair of molecules.

What might be the cause of this inadvertent splitting? First, mobile molecules are more likely to show displacements beyond the user-defined tracking window (i.e., the maximum displacement radius). Second, localizations can be missed if the intensity intermittently falls below the detection threshold due to motion blurring. Third, movement of molecules in the *z*-direction may cause them to disappear and reappear in the focal plane. Interestingly, this latter effect was shown to cause substantial undercounting of mobile tracks in experiments with large eukaryotic cells.^47^ By contrast, in thin *E. coli* cells (∼1 μm cell diameter), movement in the *z*-direction is limited, so occasional missed localizations are mostly due to changes in brightness and spot shape rather than the object being lost from the focal plane completely. These three effects may cause a genuine trajectory to be inadvertently split into multiple subtrajectories, each providing an additional measurement of the diffusion coefficient during MSD analysis. Crucially, these effects are more likely to happen to a mobile molecule compared to an immobile molecule (whose localizations are usually linked together successfully from frame-to-frame), therefore leading to an overcounting of mobile trajectories in our single-molecule tracking experiments.

**Figure 7:**
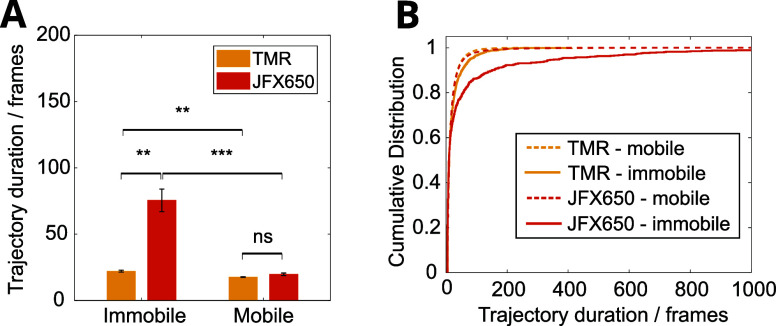
Trajectory duration dependence on molecule mobility. (A) TMR and JFX650 trajectory duration for immobile or mobile molecules (diffusion coefficient less or greater than 0.04 μm^2^s^–1^, respectively). Mean ± standard error of the mean for 3 or 6 independent repeats for TMR- and JFX650-labeled MutS-Halo, respectively. (B) Cumulative distribution of the duration of immobile (solid line) and mobile (dashed line) tracks, pooled from 3 or 6 independent repeats for TMR- and JFX650-labeled MutS-Halo, respectively.

Although this general inadvertent splitting effect is likely to be present for both TMR- and JFX650-labeled MutS-Halo molecules, we expect that the bias is amplified for photostable fluorophores like JFX650 where individual molecules that are visible for thousands of frames may inadvertently contribute a large number of high-valued diffusion coefficient measurements if accidentally split. We reasoned that this bias should be less problematic for fluorophores like TMR. This is because the frequent photoswitching of the TMR fluorophore inherently splits all trajectories into shorter segments regardless of molecular mobility, which should reduce the biases seen in the relative counting of mobile and immobile trajectories. To test whether this is true, we looked at the distribution of trajectory lengths for both TMR and JFX650. We separated the trajectories depending on whether they were associated with a mobile molecule or an immobile molecule, based on a diffusion coefficient threshold of 0.04 μm^2^s^–1^. We found that mobile trajectories had a shorter duration than immobile trajectories (Figure 7). This matches our observation that the trajectories of mobile molecules are often inadvertently split due to data analysis errors, whereas immobile molecules are reliably tracked until they permanently photobleach or until they transition to a mobile state. In fact, mobile tracks of JFX650 and TMR had the same duration, showing that the inadvertent splitting effect is limiting the lengths of the trajectories to an equal degree regardless of the fluorophore photostability. Although TMR and JFX650 both show a difference between mobile and immobile track duration, the difference is much smaller for TMR, confirming our prediction above that the bias will affect photoswitching dyes like TMR to a lesser degree. As expected, we found that immobile JFX650 trajectories lasted substantially longer than immobile TMR trajectories (75.6 vs 22.1 frames on average). This partially reflects the longer on-time of the JFX650 fluorophore. However, we note that the immobile trajectory lengths for JFX650 may also be limited here by any molecules, which transition from an immobile state to a mobile state during their trajectory, thereby also resulting in premature trajectory splitting before the JFX650 molecule permanently photobleaches.

**Figure 8:**
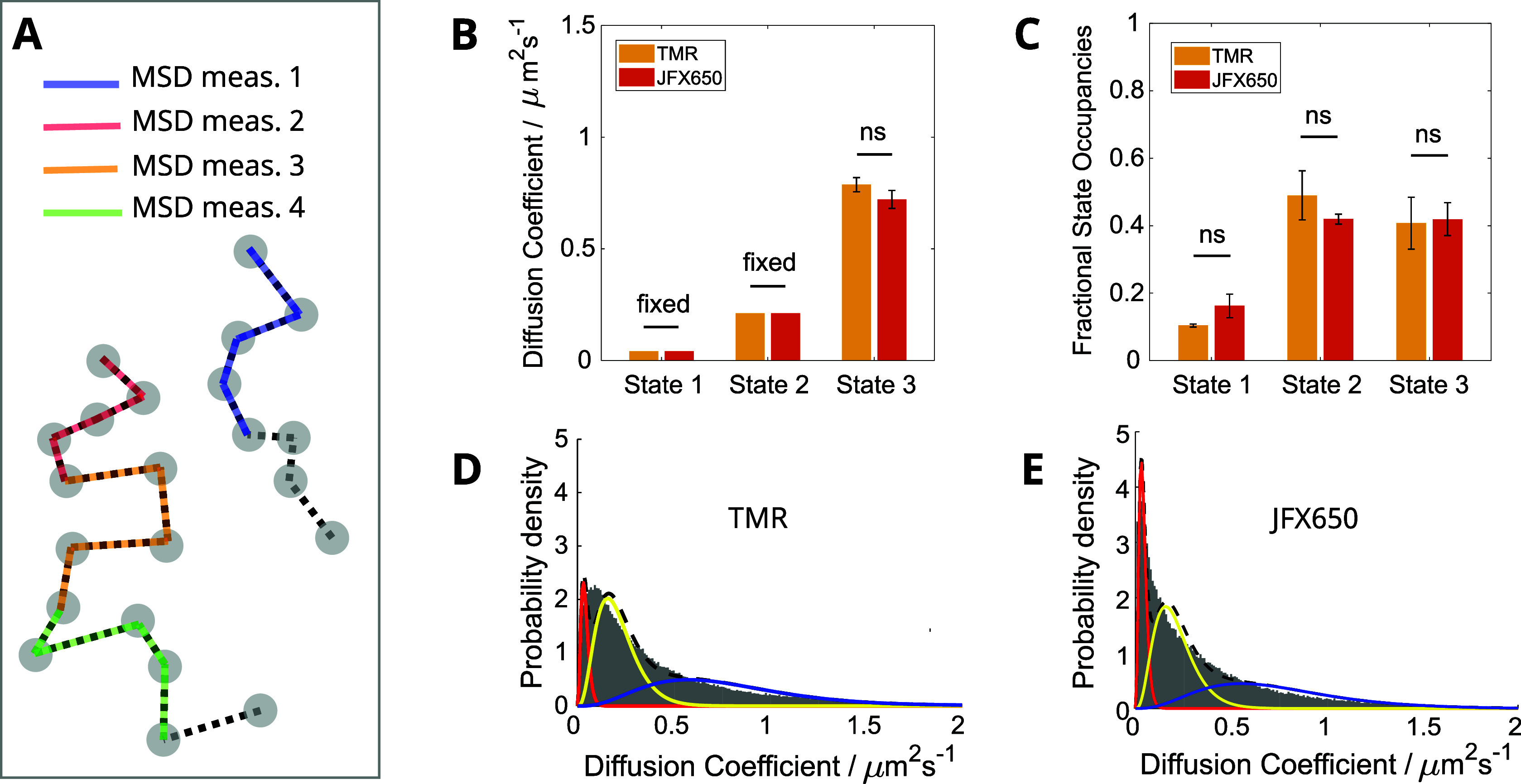
Comparison of TMR- and JFX650-labeled MutS-Halo diffusive state analysis using the MSD calculation approach with forced computational partitioning of trajectories. (A) Schematic showing the frames over which the MSD is measured for each example trajectory. Due to forced computational partitioning, multiple MSD measurements can be obtained per trajectory. (B, C) Estimated diffusion coefficients and state occupancies for the 3-state model. Error bars show the standard error of the mean across 3x (TMR) and 6x (JFX650) independent experimental repeats, and bar heights are weighted means according to the total number of trajectories per repeat. (D, E) Averaged diffusion coefficient histograms from each experiment repeat for TMR (D) and JFX650 (E). The fitted probability distribution curves are shown for the immobile (red), slow-diffusing (yellow), and fast-diffusing (blue) states.

Having identified the inadvertent splitting artifact, we tested a simple analysis solution to reduce the resulting bias in diffusive state occupancies. We used the same single-molecule tracking data that contributed to the results in Figure 5B–E, but we further partitioned the trajectories into shorter subtrajectories, each of 5 frames in length; we hereafter call this process “forced computational partitioning”. This process mimics, to some extent, the photoswitching nature of the TMR. We computed the MSD and corresponding diffusion coefficient for each of the shorter subtrajectories following forced computational partitioning; this process is depicted in Figure 8A. This forced partitioning of trajectories occurs irrespective of a molecule’s mobility and therefore aims to quash the biased overcounting of mobile molecules. As predicted, forced computational partitioning increased the estimated relative occupancies of the immobile state for both the JFX650- and TMR-labeled MutS-Halo (Figure 8B–E).

The immobile state occupancy values obtained using MSD analysis with forced computational partitioning are now more similar to those obtained by HMM analysis (*p* = 0.0479, *p* = 0.9822, for TMR and JFX650, respectively, see Supplementary Figure S5). This is reassuring, suggesting that experimental and analysis biases can be eliminated such that HMM and MSD analysis yield consistent results.

For completeness, we performed HMM analysis with the trajectories created during forced computational partitioning and found little effect on the estimated state occupancies compared to those found with the nonpartitioned trajectories (Supplementary Figure S4). HMM analysis is therefore more robust to the inadvertent splitting bias. This result is expected because HMM considers state transition probabilities on a frame-by-frame basis rather than trajectory averages.

### 3.5 JFX650 Long-Duration Single-Molecule Tracking Unlocks New Biological Insights

The lack of reversible photoswitching and high photostability of JFX650 means that only a few MutS trajectories can be recorded per cell while still retaining sparse-emitter conditions, as opposed to several hundred trajectories for TMR. This leads to an overall higher statistical uncertainty in the estimation of diffusion coefficients and state occupancies for JFX650, regardless of the analysis method used (see Supplementary Figure S6 for a comparison of the standard deviation in the immobile state frequency measurements). In principle, recording few long tracks should provide the same information as many short tracks, provided the recorded molecules represent a homogeneous sample of the overall population. This is ensured if each molecule frequently transitions between the different diffusive states. However, this is apparently not the case for MutS. Because DNA replication errors are rare and short-lived, only a small percentage of cells contain a DNA mismatch at any time (<1%^56,57^) and binding events of MutS are rare. As such, the total number of JFX650 tracks corresponding to immobile molecules is very variable between measurement repeats. Therefore, although both fluorophores provide consistent average measurements for the diffusive properties of MutS, we suggest that the increased sampling attainable from photoswitchable dyes like TMR is favorable for measuring the diffusion of a heterogeneous population of molecules.

**Figure 9:**
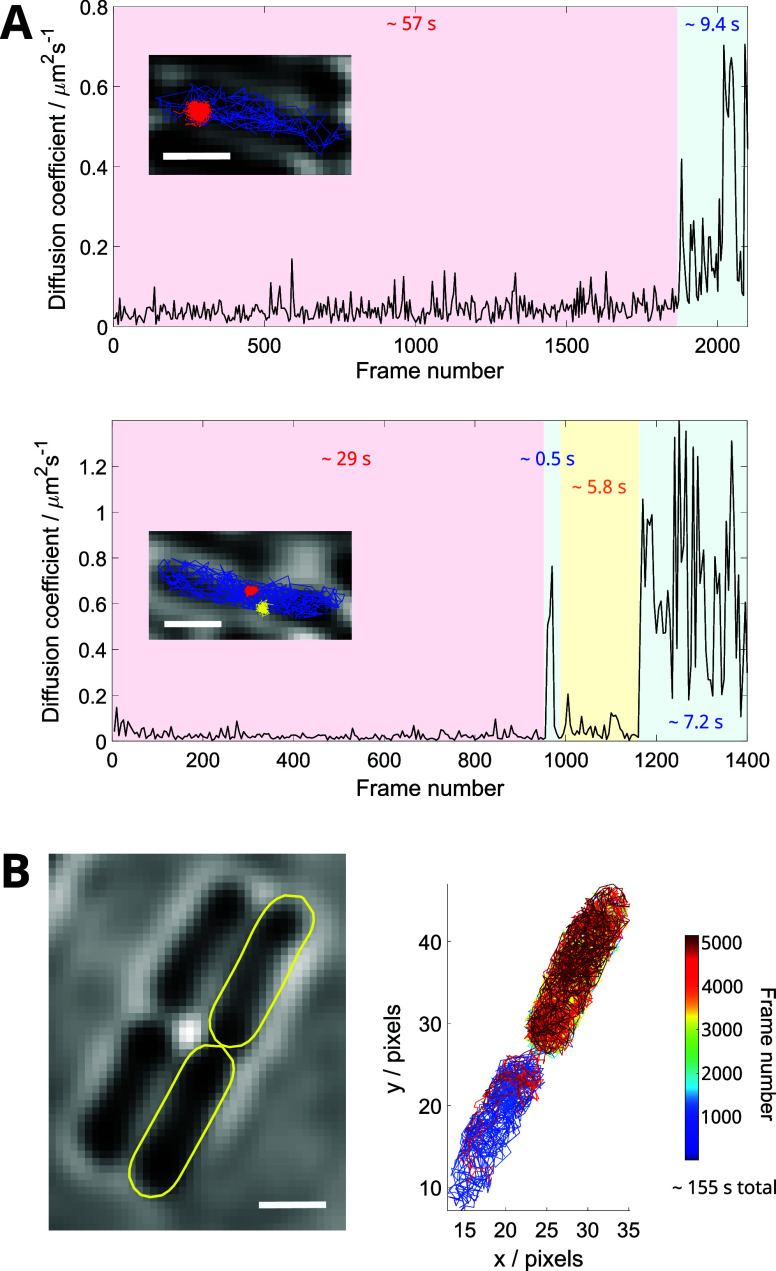
JFX650 enables prolonged single-molecule observations. (A) The frame-to-frame diffusion coefficients of two example single-molecule MutS-Halo-JFX650 tracks show long-lived binding events and transitions between mobile and immobile states. Insets show the tracks overlaid on the corresponding brightfield image, with line color corresponding to the background color of the segments in the main plot. (B) Example of a single MutS-Halo-JFX650 molecule that crossed repeatedly between daughter cells during cell division and became constrained in the top cell, likely due to septum closure. Brightfield image shows relevant cell outlines highlighted in yellow. Track color corresponds to the frame number. Scale bars are all 1 μm.

Nevertheless, there is still important novel biological information that can be extracted from single-molecule acquisitions with long-lived fluorescent dyes. For instance, we were able to directly observe the rare transitions of MutS-Halo-JFX650 between diffusive states. Figure 9A shows an example of a long-lived immobile event, lasting for 1866 frames (∼57 s), before unbinding and transitioning to a mobile state. We also observed tracks containing multiple MutS binding events or binding events that are flanked by mobile events (see also Figure 9A). In some cases, MutS-Halo-JFX650 molecules can be seen repeatedly traversing between two daughter cells in the process of division. Interestingly, in an example trajectory lasting 155 s, MutS initially moved between the two cell halves but later became constrained in the upper daughter cell, suggesting that the division septum closed during the trajectory (Figure 9B). Such measurements may therefore provide previously unattainable information about the process of cell division and the partitioning of molecules between daughter cells. We note that it is possible to prolong track durations even further using noncontinuous illumination (i.e., stroboscopic imaging)^58^ to reduce the rate of photobleaching.

## 4 Conclusions

High-speed single-molecule tracking is increasing in popularity as a method for characterizing the movement of proteins within live cells. Users of this method now have a wide choice of different imaging modalities, fluorophores and labeling strategies, and various data processing approaches. A major advance has been the development of bright and photostable synthetic dyes that can be covalently linked to genetically encoded tags, enabling single-molecule tracking on the time scale of several minutes at a temporal resolution around 10 ms. The photophysical properties of fluorophores have also been engineered, yielding photoactivatable dyes for super-resolution localization and tracking analysis, while other dyes and imaging conditions are meant to provide long-lived emission without blinking behavior.^59^ With all of these available choices, it can be difficult to decide which nuance of the single-molecule tracking method is best suited for a particular application. Furthermore, it is often unclear if the quantitative results obtained from single-molecule tracking measurements are robust to differences in the experimental conditions and analysis procedures. In a previous study, we compared the utility of photoactivatable fluorescent proteins vs HaloTag-labeled proteins for single-molecule tracking measurements.^37^ In this study, we now characterize two synthetic fluorophores with very different properties, TMR and JFX650, and tested the reliability of the two most popular data analysis approaches, HMM and MSD. Our findings provide guidance for experimental design and analysis, as well as strategies to identify and eliminate biases in data quantification.

In live *E. coli* cells, TMR and JFX650 both show bright fluorescence suitable for high-resolution localization. Whereas TMR repeatedly switches between a bright and dark state, JFX650 exhibits high photostability without switching. These differences necessitated two different experimental approaches to ensure that sparse-emitter conditions were achieved for isolating single molecules: namely, the JFX650 experiments required a lower labeling concentration compared to TMR. We found throughout our study that, where the same analysis method was used, there were no significant differences between the diffusive properties measured for TMR- and JFX650-labeled MutS-Halo. This means that, theoretically, anyone currently employing SnapTag or HaloTag labeling strategies could readily make the switch to a different fluorophore and expect the average diffusion analysis to be directly comparable across repeat experiments; this is of course, provided that similar measures to those described above are taken to adjust labeling density and fluorophore brightness. These findings are exciting as they validate the idea of cross-comparing results from studies, even where different fluorophores have been used, aiding reproducibility.

Despite the same average result being achieved for both fluorophores, we advise to exercise caution when using long-lived emitters such as JFX650 for certain single-molecule tracking experiments. We have shown that, although the choice of fluorophore makes little difference to the measurements, the choice of analysis method can make a significant difference. MSD measurements can overestimate the frequency of mobile trajectories due to inadvertent trajectory splitting. Notably, a previous study showed that the frequency of mobile trajectories is typically underestimated due to the movement of these molecules out of the focal plane.^47^ Although at first glance this opposite bias seems contradictory, our findings are complementary and explained by differences in cell morphology. In large eukaryotic cells, mobile molecules quickly disappear from the focal plane^47^ (causing undercounting), whereas in thinner bacterial cells, mobile molecules are lost only transiently and reappear multiple times (causing overcounting). This highlights the importance for careful consideration of sample-dependent biases in single-molecule tracking analysis. In our study, we have additionally shown that the overestimation of mobile trajectories is present to a greater extent in experiments with the JFX650 dye, where exceptionally long-lived fluorescence events oversample mobile compared to immobile molecules. This may become a problem where the experimental aim is to determine any changes in diffusive state occupancies of proteins of interest following genetic perturbations or cellular treatments: the lower susceptibility to the sampling bias plus the greater measurement consistency due to repeated observations of the same molecules makes short-lived photoswitchers such as TMR our fluorophore of choice for experiments of this nature. Fortunately, we have shown that computationally partitioning trajectories further into shorter trajectories of known step lengths can help to reduce this bias.

The calculations of the diffusion coefficient rely upon the assumption of Brownian motion. In reality, confinement of molecules within a cell boundary results in anomalous diffusion,^60,61^ where the MSD deviates from linear Brownian motion in a subdiffusive manner. This leads to an underestimation of the true diffusion coefficient, which particularly affects the fastest-moving molecules. In addition, large particles are affected by macromolecular crowding, exhibiting subdiffusive behavior in the densely packed cell cytoplasm. A particle’s size, shape, and charge contribute to its enrichment in, or exclusion from, different cellular compartments.^14,54,55^ Furthermore, the elongated shape of cells can lead to differences in the longitudinal and transversal mobilities of molecules, resulting in anisotropic anomalous diffusion.^62^ Overall, biochemical differences between molecules, such as their protonation, redox, and oligomeric states, together with variation in their local cellular environment, will lead to heterogeneity in the apparent diffusion coefficients across trajectories. These effects may contribute to the observed broad distribution of diffusion coefficients for MutS-Halo, particularly for MSD analysis. Newer tools (utilizing for example Bayesian,^63,64^ machine-learning^65,66^ and deep-learning based approaches^67−70^) present an attractive analysis option, as they aim to account for anomalous diffusion effects; more so because they display improved performance for the case of long-lived trajectories,^66^ which we have shown are now achievable in a biological context, here using JFX650. However, we note that such tools will also be subject to the overcounting bias that we have described here, since they are also applied to prelinked trajectories (where the localization and linking steps have already been carried out). As such, we note that a promising new approach bypasses tracking entirely and was shown to provide reliable estimation of diffusion coefficients and state occupancies even at high-emitter densities.^71^

Even though long-lived fluorescent emitters such as JFX650 are not optimal for all experiment types and analysis methods, we have shown that they can provide valuable insights in other flavors of single-molecule tracking studies. The ability to track the same individual molecule at high temporal resolution for many minutes means that we are more likely to directly see transitions between diffusive states. Instead of inferring average transition probabilities and average state lifetimes, there is an opportunity to compute these values directly from individual events and therefore also measure their variability. Taking MutS as an example, we can then begin to explore the spatial and temporal factors that are important for allowing MutS to become immobilized on DNA, and thereby understand the underlying mechanisms of DNA mismatch recognition within cells. Experiments of this nature are sure to be where new long-lived emitters such as JFX650 will excel.

## Supporting Information Available

Further details regarding sample preparation, data acquisition, and data analysis; quantification of localization error from TMR and JFX650; confirmation of dead *E. coli* cells in microscopy images and details of methods used for excluding them from single-molecule tracking analysis; varying the maximum number of allowable hidden states for MutS-Halo single-molecule tracking data during vbSPT analysis; vbSPT analysis of partitioned trajectories; and variability of measurements of the immobile population (PDF)
