# Supplementary material for: Robust Quantification of Live-Cell Single-Molecule Tracking Data for Fluorophores with Different Photophysical Properties
Source: J Phys Chem B. 2024 Jun 11;128(30):7291–303. doi: 10.1021/acs.jpcb.4c01454 (PMC11301680; doi:10.1021/acs.jpcb.4c01454)
Supplement: Supplementary file 1 — jp4c01454_si_001.pdf [file jp4c01454_si_001.pdf]

Robust Quantification Of Live-Cell Single-Molecule Tracking Data For Fluorophores With Different Photophysical Properties

Supplementary Information

Authors: Amy N. Moores[1], Stephan Uphoff[2]*

[1,2] Department of Biochemistry, University of Oxford, South Parks Rd, Oxford, OX1 3QU, UK

*correspondence: stephan.uphoff@bioch.ox.ac.uk

S.1. Further details regarding sample preparation, data acquisition and data analysis

S.1.1. The construction of *E. coli* cells expressing MutS-Halo

The bacterial strain used for this study is derived from *E. coli* AB1157. We replaced the endogenous *mutS* gene with a HaloTag fusion, which enables covalent bonding with cell-permeable fluorophores such as the tetramethylrhodamine (TMR) and JFX650 used in these experiments. The MutS-Halo fusion was generated by Lambda Red recombination[1]. We inserted the HaloTag[2] sequence and a flexible 11-amino acid linker at the C terminus of the endogenous mutS gene, followed by a kanamycin resistance cassette. The allele was moved into the wild-type AB1157 strain by P1 phage transduction. The kanamycin resistance cassette was removed by expressing Flp recombinase from plasmid pCP20. Growing cells at 37 °C cured the temperature-sensitive pCP20 plasmid to produce the strain used for all experiments here. The presence of the gene fusion was confirmed by colony PCR and through fluorescence imaging for both the intermediate kanamycin-resistant strain and for the subsequent strain where kanamycin resistance has been removed. Full functionality of the MutS-Halo fusion protein was confirmed by measuring the frequency of spontaneous rifampicin resistance mutations.

S.1.2. Recipe for minimal M9 glucose growth and imaging medium

The following are added to a small volume (approximately 100 mL, to prevent precipitation) of MilliQ water in an autoclaved glass bottle: 500 µL 100 mM $CaCl_2$, 1 mL 1M $MgSO_4$, 100 mL 5x M9 minimal salts (*Sigma-Aldrich*), 10 mL 50x MEM amino acids ([-] L-Glutamine, *Gibco*), 5 mL 10 mg/ml L-Proline (dissolved in MilliQ water), 50 µL 0.5% Thiamine, 5 mL 20 % Glucose; this is then topped up with MilliQ water to make a total volume of 500 mL M9 medium. The medium is filtered using a Nalgene filter and a vacuum pump, and then stored at 4 °C. M9 media is warmed to room temperature prior to use.

S.1.3. Microscope Configuration

For all single-molecule tracking experiments, we use a custom-made inverted optical microscope consisting of: multilaser engine (*iChrome MLE, Toptica Photonics*) capable of λ = 405, 488, 561, 640 nm emission, EMCCD camera (*iXon Ultra 897, Andor*), and a hardware control unit (*TG-1000, ASI Applied Scientific Instrumentation*) which controls the following *ASI* components: XY stage (for

sample mounting and translating), Z stage and piezo stage (for relative sample stage and objective lens movement), dichroic filter slider, emission filter wheel, LED condenser on/off toggle. Objective lens used: Nikon Plan Apo λ, 100x / 1.45 oil. We use a quadband (*ZT405/488/561/640rpc, Chroma*) dichroic mirror for excitation of the sample and transmission of fluorescence to the camera. For imaging in each of the 405, 488, 561, 640 nm channels respectively, the filter wheel contains emission filters with bandwidths: 425-475 nm, 500-550 nm (*both from ZET405/488/561m, Chroma*), 575 nm longpass (*ET575, Chroma*), 655 nm longpass (*ET655, Chroma*).

The microscope has total internal reflection fluorescence (TIRF) illumination mode capabilities via a translatable stage (*MB1530F/M, Thorlabs*) onto which a series of lenses are mounted, such that movement of the stage translates the position that the laser is incident at the back focal plane of the objective; this stage is translated by a motorized actuator (*z812, Thorlabs*) and controller (*Kinesis KDC101, Thorlabs*).

For the single-molecule tracking of MutS-Halo in this study, we use an exposure time of 30 ms. Accounting for the readout time per frame, the total capture duration between subsequent frames is 0.030475 s, which is used for single-molecule tracking analysis. We use a frame size of 300 (H) x 512 pixels (W), where each pixel has a physical size of 16 µm for the Andor camera used; accounting for system magnification, our pixel resolution is 107 nm. EMCCD gain is also used for all fluorescence imaging and is set to a value of 300x. For camera control, we use Solis software (*Andor*). For controlling all other microscope components, we use micro-manager open source software[3].

S.1.4. Data analysis: probability density function for histogram-fitting

To estimate the average diffusion coefficient D of diffusive states and their relative populations, we fit histograms of apparent diffusion coefficient ($D*$) measurements to a mixture of probability density functions[4,5].

The distribution of $D*$ values takes the form of a Gamma distribution:

$$f_D * (x; D, n) = \frac{(^n/_D)^n x^{n-1} e^{-nx/_D}}{(n-1)!}$$

Where n is the number of steps the mean-square displacement (MSD) is being calculated over (here we use 4-steps), D is the average diffusion coefficient, and x is the number of individual measurements made.

We believe there to be three diffusive states present in our single-molecule tracking data for MutS-Halo, so the distribution of $D*$ values is therefore fit to a summation of three probability density functions, where D_1, D_2 and D_3 are the average diffusion coefficients of states 1, 2, and 3 respectively:

$$f_D * (x; D, n) = \frac{A_1 \left(^n/_{D_1}\right)^n x^{n-1} e^{-nx/_{D_1}}}{(n-1)!} + \frac{A_2 \left(^n/_{D_2}\right)^n x^{n-1} e^{-nx/_{D_2}}}{(n-1)!} + \frac{A_3 \left(^n/_{D_3}\right)^n x^{n-1} e^{-nx/_{D_3}}}{(n-1)!}$$

and where: $A_1 + A_2 + A_3 = 1$, corresponding to the relative occupancies of each diffusive state.

S.2. Quantification of localization error from TMR and JFX650

The localization error can be inferred from measurements of the apparent diffusion coefficient of molecules which are in an immobile state.

The apparent diffusion coefficient is calculated as follows:

$$D* = \frac{MSD}{4\,\Delta T} - \frac{\sigma loc^2}{\Delta T}$$

where *MSD* is the mean-square displacement, *ΔT* is the time between successive frames, and σ_{loc} is the localization error. We describe the use of vbSPT to determine the average diffusion coefficients of the three diffusive states of TMR- and JFX-labelled MutS-Halo (main text, Figure 4). Knowing that the MSD of immobile molecules is approximately zero, we can calculate the localization error from D* measurements of the immobile diffusive state (state 1 in our study). We see no significant differences between the estimated mean localization errors for TMR- and JFX650-labelled MutS (Figure S1).

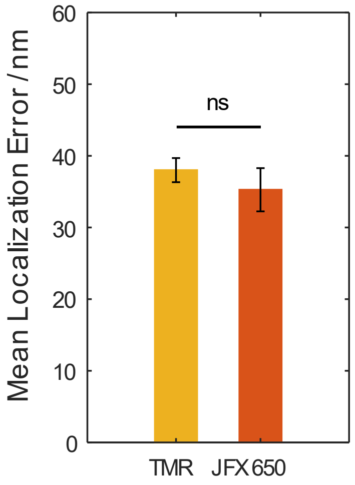

Figure S1: The mean localization error for MutS-Halo-TMR and MutS-Halo-JFX650, inferred from vbSPT measurements of the immobile state diffusion coefficient. Bar heights show the mean values and errorbars show the standard error of the mean from 3 and 6 independent experimental repeats for TMR and JFX650, respectively.

S.3. Confirmation of dead *E. coli* cells in microscopy images and details of methods used for excluding them from single-molecule tracking analysis

During single-molecule tracking experiments, we noticed a subpopulation of cells in the sample which took longer than average to photobleach (i.e. longer than 500 frames, see main text, Figure 2 for general photobleaching characteristics of TMR). We hypothesised that these cells were potentially dead, and should be excluded from analysis.

To confirm this, we performed a dual-labelling experiment to add SYTOX™ Green nucleic acid stain (*Invitrogen*) to *E. coli* cells alongside the TMR-labelling of MutS-Halo; we followed the same sample preparation protocol as described in the main text section 2.1, except that SYTOX was added to the cell culture at the same time as TMR to a final concentration of 5 nM. To image, we used the same procedure as for the single-molecule tracking protocol (see main text, methods), with an additional imaging step. Briefly, we used an exposure time of 100 ms, whilst keeping the 488 nm laser at very low power density (< 1 Wcm^{-2}). We acquired a short movie (1000 frames) from which a mean intensity projection could be calculated through the 1000 frames (example shown in Figure S2a), indicating the locations of SYTOX-positive (dead) cells. To test for correlation with TMR photobleaching rate, we acquired a movie of 1000 frames from laser turn-on (in the 561 nm channel), and calculated a median intensity projection through the 1000 frames (example shown in Figure 2b). Comparing figures S2a and S2b, we see that SYTOX-positive cells correlate overwhelmingly to the cells in which TMR fluorescence bleaches slowly, and we therefore use this median intensity projection method to exclude these cells from our single-molecule tracking analysis, without the need for SYTOX labelling for every experiment.

Similarly to the TMR experiments, we also find a subpopulation of dead cells in the JFX650 experiments (Fig S2c). We correlated SYTOX-positive cells in this case to cells which were still fluorescing in the 640 nm channel at the end of our usual 5000-frame JFX650 single-molecule tracking acquisition. To exclude these cells from analysis, we therefore generate a mean intensity projection of the final 500 frames of our single-molecule tracking movie (see example in Fig. S2d), highlighting the locations of these cells without the need for SYTOX staining in every experiment.

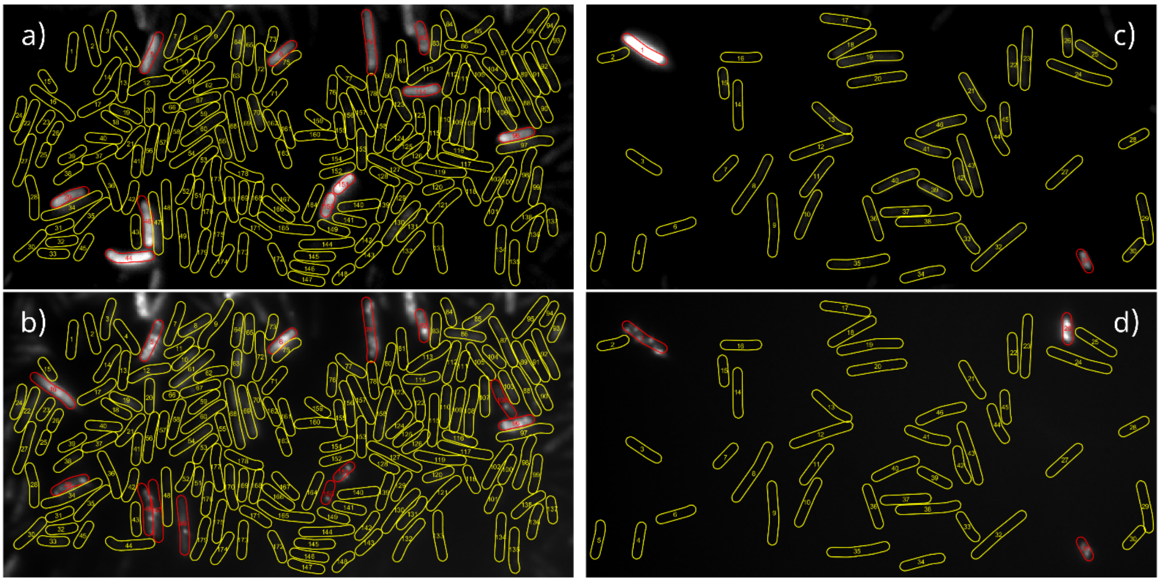

Figure S2: Correlation of SYTOX-positive (dead) cells to TMR- and JFX650-labelled "slow-to-bleach" cells. **a)** Example image of *E. coli* cells which have been labelled with SYTOX Green. Numbered cell outlines are shown in yellow and SYTOX-positive cells are highlighted in red. **b)** The same cells as in a) have been labelled with TMR. Image shows a median intensity projection of the first 1000 frames from laser turn-on in the TMR channel. "Slow-to-bleach" cells are highlighted in red, with normal cells in yellow. **c)** Example image of *E. coli* cells which have been labelled with SYTOX Green. SYTOX-positive cells shown in red. **d)** The same cells as in c) have been labelled with JFX650. Image shows a mean intensity projection of the final 500 frames of the single-molecule acquisition in the JFX650 channel, in which "slow-to-bleach" cells are highlighted in red.

S.4. Varying the maximum number of allowable hidden states for MutS-Halo single-molecule tracking data during vbSPT analysis

For all analysis tools used in this study, a user-defined value for the number of diffusive states is required. To aid our estimation of this value, we used the Hidden Markov Modelling tool vbSPT[6].

For each single-molecule tracking session of MutS-Halo-TMR and MutS-Halo-JFX650, we analysed all trajectories using vbSPT, storing the estimated diffusion coefficients and state occupancies. We repeated this process 5 times for each dataset, varying the maximum number of diffusive states from 2 up to 6. The mean diffusion coefficient and state occupancy values of this iterative analysis across all experimental repeats are shown in Figure S3.

We found broadly that the 3-state model encompasses well the estimates that were found with larger numbers of states, aligning well with the estimates from the 6-state model (which is likely to overfit our data). This also supports observations from our microscopy data, where we find evidence of at least 3 clear diffusive states (main text, Figure 3).

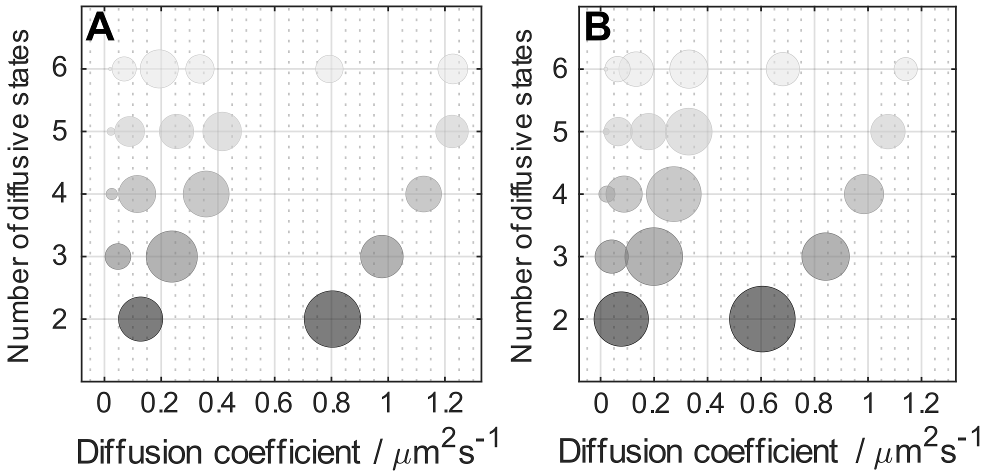

Figure S3: The mean vbSPT outputs in response to varying the maximum number of allowable diffusive states, measured across 3 and 6 independent single-molecule tracking experiments for MutS-Halo-TMR (A) and MutS-Halo-JFX (B) respectively. Position of the circles along the x-axis shows the estimated diffusion coefficient for each state, and the size of the circle is proportional to the state occupancy.

S.5. vbSPT analysis of partitioned trajectories

In our study, we tested how partitioning the single-molecule trajectories affects the diffusion analysis when using the MSD calculation method. For completeness, we tested the HMM analysis method to see whether the result would differ depending on whether non-partitioned or partitioned trajectories were used (Figure S4). Performing vbSPT analysis on tracks partitioned into 5-frame sections produced similar outputs to the non-partitioned tracks (shown in Figure 4, main text), indicating that HMM analysis is less sensitive to biases caused by trajectory length or number.

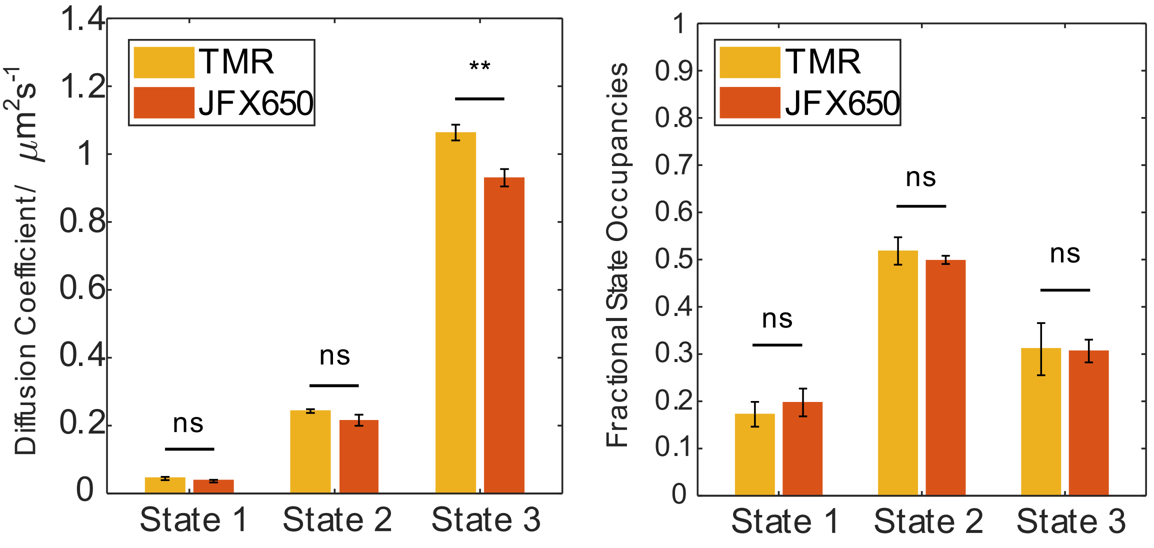

Figure S4: A comparison of TMR and JFX650-labelled MutS-Halo diffusive state analysis using HMM, where the input trajectories have been subjected to imposed partitioning. Shown are the diffusion coefficient values and the state occupancies for the 3-state model. Error bars show the standard error of the mean across 3x (TMR) and 6x (JFX650) independent experimental repeats and bar heights are weighted means according to the total number of trajectories per repeat.

S.6. Variability of measurements of the immobile population

The population of the immobile state (state 1) is particularly significant from a biological point of view, as it signifies the frequency of DNA mismatches and the time that MutS molecules dwell at mismatch repair sites. We compared values of the state 1 occupancy measurements across the different analysis methods used (Figure S5). We find that the MSD method provides a significantly lower immobile state occupancy value than the HMM analysis method, which we attribute to the inadvertent splitting of mobile trajectories.

We also calculated the standard deviation of the immobile population measurements across all experimental repeats for each condition: TMR or JFX650-labelled MutS-Halo for each analysis method respectively (see Figure S6). Overall, standard deviations are larger for JFX650 compared to TMR across all analysis methods, suggesting increased measurement variability between experiments.

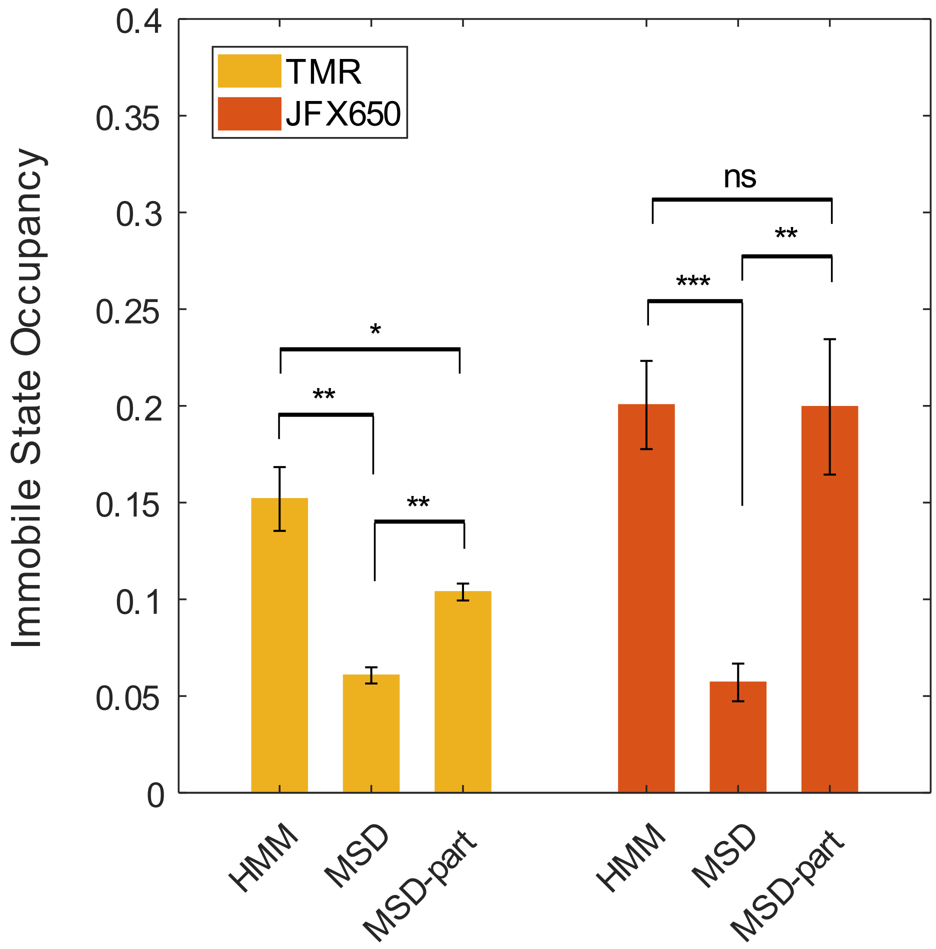

Figure S5: A comparison of the measured occupancies of state 1 (immobile state) for TMR and JFX650-labelled MutS-Halo, depending on analysis method used: HMM, MSD, and MSD on partitioned trajectories (MSD-part); bar heights show the mean values of the state 1 occupancies measured across 3 and 6 experimental repeats for TMR and JFX650 respectively, and error bars show the standard error of the mean.

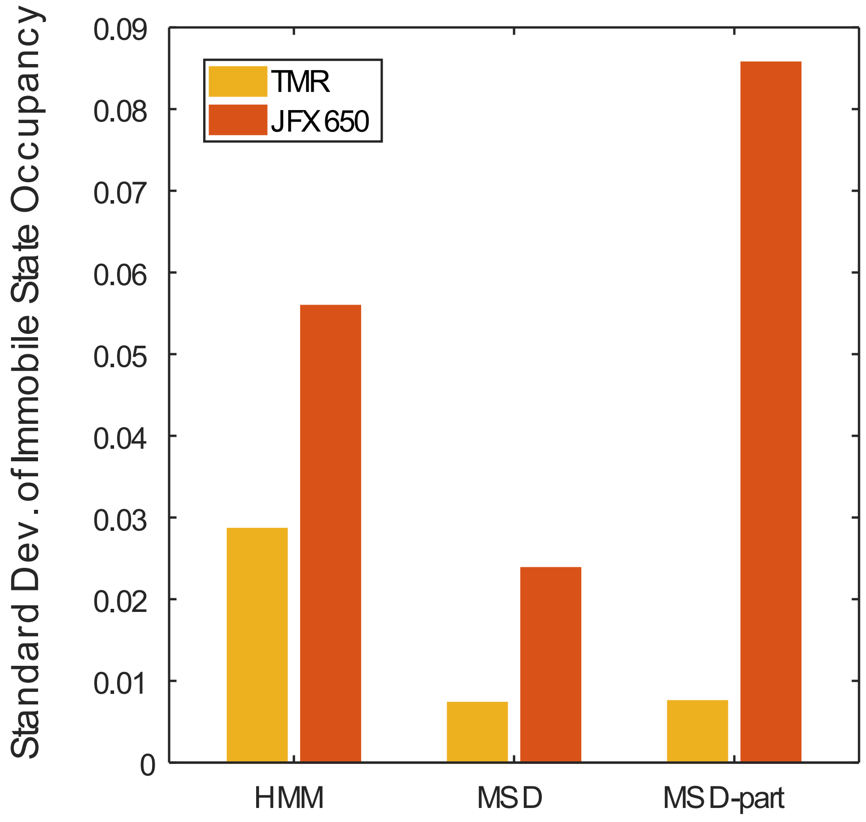

Figure S6: Standard deviation across experimental repeats for measurements of state 1 occupancy (immobile state) for TMR and JFX650-labelled MutS-Halo. Values are shown separately for the three analysis methods used on our datasets: HMM, MSD and MSD on partitioned trajectories (MSD-part).

References

1. Datsenko, K. A. & Wanner, B. L. One-step inactivation of chromosomal genes in Escherichia coli K-12 using PCR products. *Proc. Natl. Acad. Sci. U. S. A.* **97**, 6640–6645 (2000).
2. Los, G. V. *et al.* HaloTag: A Novel Protein Labeling Technology for Cell Imaging and Protein Analysis. *ACS Chem. Biol.* **3**, 34 (2024).
3. Edelstein, A., Amodaj, N., Hoover, K., Vale, R. & Stuurman, N. Computer control of microscopes using µmanager. *Curr. Protoc. Mol. Biol.* (2010) doi:10.1002/0471142727.MB1420S92.
4. Saxton, M. J. Single-particle tracking: the distribution of diffusion coefficients. *Biophys. J.* **72**, 1744–1753 (1997).
5. Stracy, M. *et al.* Live-cell superresolution microscopy reveals the organization of RNA polymerase in the bacterial nucleoid. *Proc. Natl. Acad. Sci.* **112**, E4390–E4399 (2015).
6. Persson, F., Lindén, M., Unoson, C. & Elf, J. Extracting intracellular diffusive states and transition rates from single-molecule tracking data. *Nat. Methods* **10**, 265–269 (2013).
